# Sustainable source of omega-3 eicosapentaenoic acid from metabolically engineered *Yarrowia lipolytica*: from fundamental research to commercial production

**DOI:** 10.1007/s00253-014-6318-y

**Published:** 2015-01-08

**Authors:** Dongming Xie, Ethel N. Jackson, Quinn Zhu

**Affiliations:** Biotechnology, Central Research and Development, E.I. du Pont de Nemours and Company, Wilmington, DE USA

**Keywords:** *Yarrowia lipolytica*, Omega-3 fatty acid, Metabolic engineering, Fermentation, Commercialization

## Abstract

The omega-3 fatty acids, cis-5, 8, 11, 14, and 17-eicosapentaenoic acid (C20:5; EPA) and cis-4, 7, 10, 13, 16, and 19-docosahexaenoic acid (C22:6; DHA), have wide-ranging benefits in improving heart health, immune function, mental health, and infant cognitive development. Currently, the major source for EPA and DHA is from fish oil, and a minor source of DHA is from microalgae. With the increased demand for EPA and DHA, DuPont has developed a clean and sustainable source of the omega-3 fatty acid EPA through fermentation using metabolically engineered strains of *Yarrowia lipolytica*. In this mini-review, we will focus on DuPont’s technology for EPA production. Specifically, EPA biosynthetic and supporting pathways have been introduced into the oleaginous yeast to synthesize and accumulate EPA under fermentation conditions. This *Yarrowia* platform can also produce tailored omega-3 (EPA, DHA) and/or omega-6 (ARA, GLA) fatty acid mixtures in the cellular lipid profiles. Fundamental research such as metabolic engineering for strain construction, high-throughput screening for strain selection, fermentation process development, and process scale-up were all needed to achieve the high levels of EPA titer, rate, and yield required for commercial application. Here, we summarize how we have combined the fundamental bioscience and the industrial engineering skills to achieve large-scale production of *Yarrowia* biomass containing high amounts of EPA, which led to two commercial products, New Harvest™ EPA oil and Verlasso® salmon.

## Introduction

Omega-3 fatty acids refer to the long-chain polyunsaturated fatty acids (LCPUFA) with the first C = C double bond at the n-3 position, i.e., the third carbon from the methyl end of the carbon chain. There have been many clinical studies showing a wide range of health benefits from the omega-3 LCPUFAs, especially the eicosapentaenoic acid (C20:5; EPA) and docosahexaenoic acid (C22:6; DHA) (Martins et al. 2013; Chacon-Lee and Gonzalez-Marino 2010; Kapoor and Patil 2011; Calder 2006). In general, it is believed that EPA is able to improve cardiovascular health, mental health, and immune function, while DHA is able to improve mental health and infant cognitive development. The Japan EPA Lipid Intervention Study (JELIS) showed that EPA is a promising treatment for prevention of major coronary events (Yokoyama et al. 2007). The AMR101 study also showed that pure EPA fatty acid significantly reduced triglyceride levels in adult patients with severe hypertriglyceridemia (Ballantyne et al. 2012). The human body can only inefficiently synthesize the EPA and DHA from omega-3 alpha-linolenic acid (C18:3; ALA) but cannot de novo synthesize them (Kapoor and Patil 2011). EPA and DHA in our bodies are largely from our foods, especially cold-water oceanic fishes (Martins et al. 2013).

**Fig. 1:**
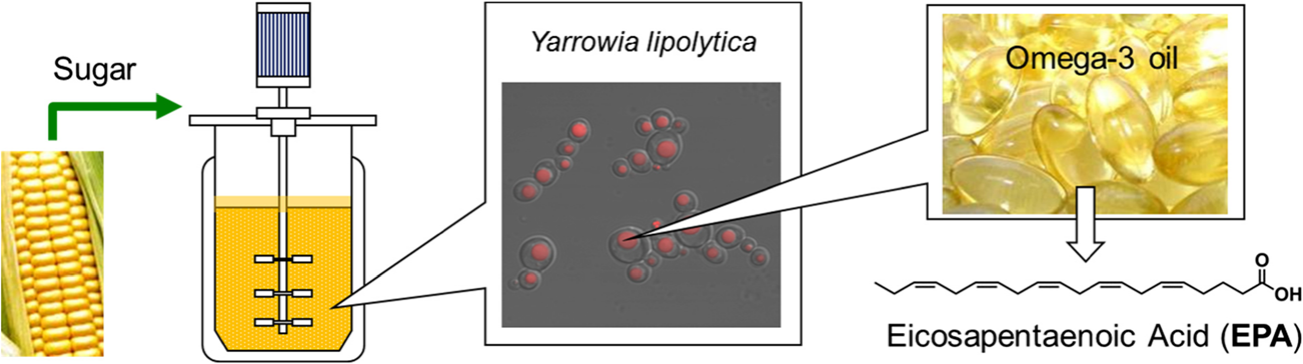
Fermentation production of eicosapentaenoic acid (EPA, C20:5 n-3) from sugar by metabolically engineered *Yarrowia lipolytica* strains

EPA and DHA are synthesized de novo in marine microorganisms and phytoplankton. Some ocean fishes (e.g., wild salmon, Pacific sardine) can accumulate significant amounts of EPA and DHA by eating microalgae cells in the ocean. Fish oil is the main source of EPA and DHA; however, its availability and sustainability have been questioned due to overfishing and contamination in the ocean environment. To overcome this limitation, biotechnology industries started to produce DHA directly from microalgae in large-scale fermentation process (Kyle 2001). However, there is no large scale land-based EPA production from wild-type organisms, because EPA productivity is too low to meet commercial targets. Consequently, DuPont initiated a research program to develop a sustainable EPA source by metabolic engineering of *Yarrowia lipolytica* (Fig. 1). We now have successfully demonstrated that the engineered *Y. lipolytica* strains can produce various omega-3 and omega-6 fatty acids. Our first targeted product for commercialization was EPA due to its unique health benefits and the lack of a land-based sustainable supply.

This mini-review summarizes DuPont’s results in both metabolic engineering research and fermentation process development for commercial production of EPA. The engineered Gen I strain Y4305 (Xue et al. 2013a) produced EPA at more than 15 % of its dry cell weight (DCW); the Gen II strain Z1978 (Hong et al. 2011a) produced EPA at more than 20 % of its DCW, the Gen III HP strain Z5567 (Hong et al. 2011a) produced EPA at more than 25 % of its DCW. The purified lipids from EPA producing strains have been used to develop a commercial product, New Harvest™ EPA oil, for a human nutritional supplement. The high-EPA biomass of these strains has also been used to raise Verlasso®, a sustainably farmed salmon. This is a good example of a yeast metabolically engineered to produce a commercial product to replace a fish-derived product. Our work has paved the way for further improvement of EPA production strains and development of strains with desired fatty acid compositions for specific applications. This advanced *Yarrowia* biotechnology platform can also be used for production of other high-value products.

## *Y. lipolytica* is a safe and productive host for EPA production

Appropriate host selection is a pre-request for the success of this project. The number one selection guideline is the safety of the organism. *Y. lipolytica* is found primarily in foods with high proportions of fat and/or protein, particularly in (fermented) dairy products and meat. Extensive research and analyses demonstrated that *Y. lipolytica* is a safe organism to be used for industrial applications (Groenewald et al. 2014). *Y. lipolytica* was first used to produce single cell protein using cheap and abundant *n*-paraffins as the sole carbon source for animal feeds under the trade name of *Toprina* (Ratledge 2005); it was also classified as “Generally Recognized as Safe (GRAS)” for commercial production of food grade citric acid (US Food and Drug Administration list of microbial-derived ingredients approved for use in food; Title 21, Part 173, Sec. 165). Other applications include production of erythritol, wax esters, 2-ketoglutaric, 2-hydroxyglutaric, and isopropylmalic acids and secretion of heterologous proteins, including several food enzymes (Ratledge 2005; Groenewald et al. 2014).

*Y. lipolytica* has an established history of robust fermentation performance. The cell density can reach more than 100 g DCW/L with carbohydrates such as glucose, fructose, glycerol, or fatty acids as sole carbon source. Most *Y. lipolytica* strains are haploid (Barth and Gaillardin 1997), but can also exist in diploid form. Depending on growth conditions, *Y. lipolytica* cells can differentiate into yeast, pseudomycelium, and true mycelial forms (Pérez-Campo and Domínguez 2001; Szabo and Stofaníková 2002). *Y. lipolytica* has a metabolism that is well suited to fatty acid production and lipid accumulation (Blank et al. 2005; Nicaud 2012; Tai and Stephanopoulos 2013); it has also been used as host organism for sustainable production of biodiesel, functional dietary lipid compounds, and other value-added compounds (Beopoulos et al. 2009 and 2010; Abghari and Chen 2014).

Some *Y. lipolytica* strains are oleaginous organisms that can accumulate up to more than 30 % DCW as storage triglycerides (TAG) under the condition of nitrogen starvation and glucose excess. Although the central carbon metabolism of *Y. lipolytica* is similar to other yeasts, it has significant regulatory differences. It also has high flux for the pentose phosphate pathway that generates cofactor NADPH to support lipid biosynthesis (Blank et al. 2005). The lipid from glucose-grown cells is comprised mainly of TAG in which oleic acid (C18:1 n-9) and linoleic acid (LA, C18:2 n-6) are the two major fatty acids (Xue et al. 2013a).

There are six chromosomes in *Y. lipolytica.* A complete genome sequence of strain CLIB122 has been published (Dujon et al. 2004). It has a total of about 20 Mb DNA that encodes about 6,500 genes. There is no extra-chromosomal plasmid discovered in wild-type strains. Genetic transformation occurs when exogenous DNA integrates into the genome by homologous and nonhomologous recombination. *Y. lipolytica* has been used as a model system for studying hydrophobic substrate utilization, peroxisome biogenesis, lipid metabolism, and bio-lipid production (Nicaud 2012; Tai and Stephanopoulos 2013). It is easy to develop auxotrophic mutants for *Y. lipolytica*. Transformants can be selected by complementation of auxotrophic mutations, and the use of antibiotic resistance genes as selectable markers is not required. The auxotrophic markers most commonly used are the *LYS5* gene coding for saccharopine dehydrogenase (Xuan et al. 1988), the *LEU2* gene coding for beta-isopropylmalate dehydrogenase (Davidow et al. 1987a, b), and the *URA3* gene encoding for orotidine 5′-monophosphate decarboxylase (Mauersberger et al. 2001). The counter selection system of the *URA3* gene and 5-fluoroorotic acid (5-FOA) allows multiple rounds of integration of functional genes into the *Y. lipolytica* genome thereby to introduce many copies of foreign genes (Barth and Gaillardin 1996; Zhu et al. 2010). In the last 30 years, *Y. lipolytica* is one of the most studied unconventional yeasts (Nicaud 2012). There is extensive knowledge accumulated on its genetics, molecular biology, and physiology. Most of these studies suggest that *Y. lipolytica* is a good model system not only for basic scientific research but also for industrial applications.

We collected and screened over 40 different *Y. lipolytica* strains from various public depositories all over the world for their fermentation performances and ability to accumulate omega-3 fatty acids such as EPA and DHA when these fatty acids are fed as substrates. After a careful execution of statistically designed experiments, the strain American Type Culture Collection (ATCC) #20362 achieved our fermentation performance targets: DCW greater than 100 g/L, lipid content greater than 30 % DCW, and lipid productivity greater than 1 g/L/h. We then selected ATCC #20362 strain for pathway engineering. The genome sequence of strain ATCC #20362 has more than 99 % identity with the genome sequence of strain CLIB122 (www.genolevures.org/yali.html). Like French strain W29 and German strain H222, strain ATCC #20362 does not contain retrotransposon-like element (Ylt1) that exists in strain CLIB122 and some American strains (Schmid-Berger et al. 1994; Mauersberger et al. 2001).

## EPA biosynthetic pathways

**Fig. 2:**
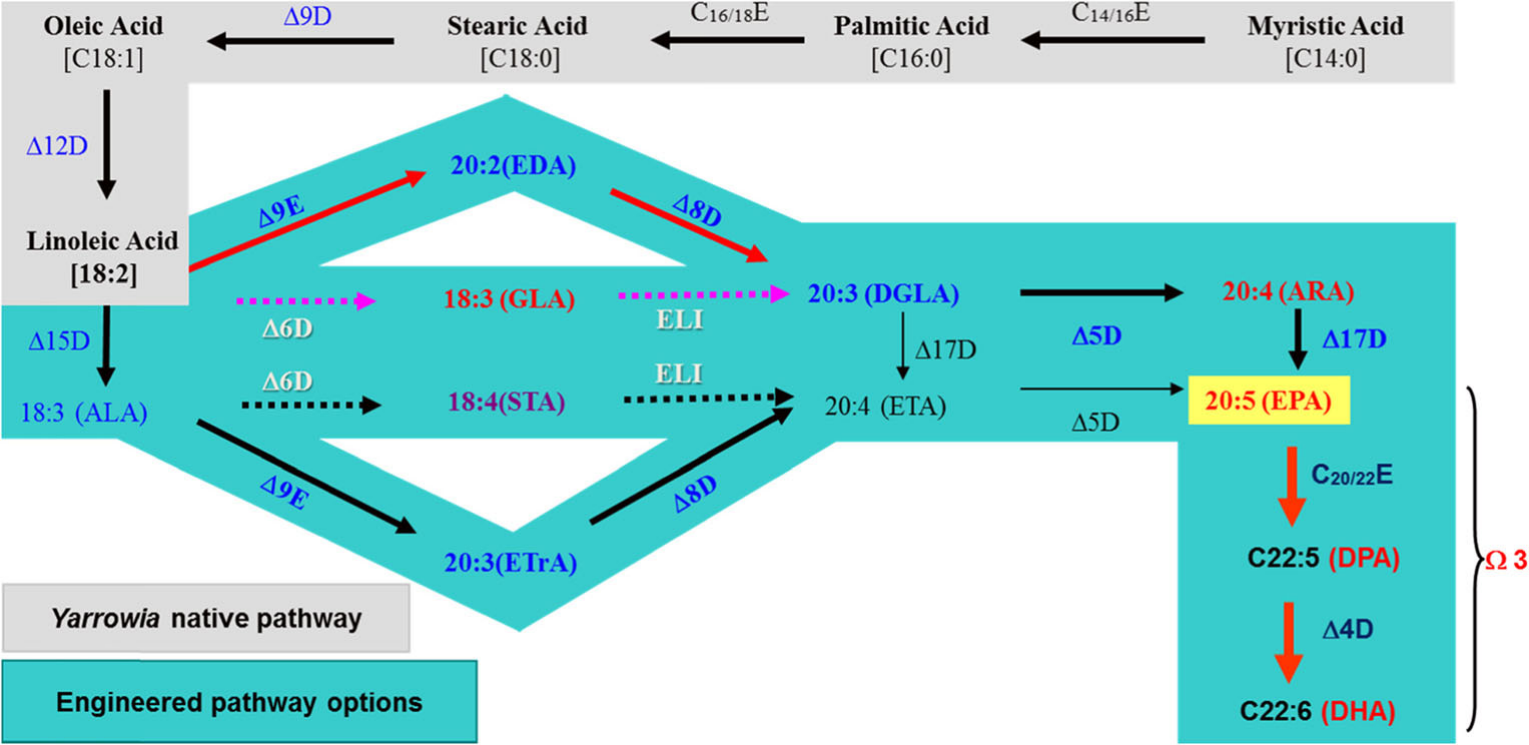
Metabolic engineering in *Yarrowia lipolytica* for omega-3 EPA and DHA production. The native fatty acid pathway is indicated in *gray* and the engineered pathway for omega-3 EPA and DHA production is indicated in *green* (color figure online)

Wild-type *Y. lipolytica* does not make any omega-3 fatty acids. The fatty acid profile of the wild-type strain ATCC #20362 (Zhu et al. 2010; Xue et al. 2013a) shows that it can synthesize linoleic acid (LA, C18:2 n-6). There are different published biosynthetic routes to make EPA, the anaerobic polyketide synthase pathway (Metz et al. 2001) or an aerobic desaturase and elongase pathway (Meesapyodsuk and Qiu 2012). The microalgae *Crypthecodinium cohnii* and *Schizochytrium* sp. used for DHA commercial production use the polyketide synthase pathway. Many microalgae and some marine bacteria also use the polyketide synthase pathway to synthesize EPA (Metz et al. 2001; Wen and Chen 2005). However, the rate, titer, and yield from these organisms could not meet the requirement for commercial production. The aerobic pathway (Fig. 2) can be further classified into a ∆6-desaturase pathway (the ∆6 pathway found in algae, mosses, fungi, and others) or a ∆9-elongase and ∆8-desaturase (Wallis and Browse 1999) pathways (the ∆9 pathway). The ∆9 pathway (Sayanova and Napier 2004) has been found in some species from Prymnesiophyceae (*Pavlova*, *Isochrysis*), Acanthamoebae (e.g., *Acanthamoeba*) and Euglenophyceae (e.g., *Euglena*).

The differences between the ∆6 and ∆9 pathways are the first two steps. In the ∆6 pathway, the first step is the ∆6 desaturase to covert the LA and/or ALA to gamma-linolenic acid (GLA, 18:3 n-6) and/or stearidonic acid (STA, 18:4 n-3). The second step is the C_18/20_ elongase to convert the GLA and/or STA to dihomo-gamma-linoleic acid (DGLA, 20:3 n-6) and/or eicosatetraenoic acid (ETA, 20:4 n-3). In the ∆9 pathway, the first step is the ∆9 elongase to convert LA and/or ALA to eicosadienoic acid (EDA, 20:2 n-6) and/or eicosatrienoic acid (ETrA: 20:3 n-3). The second step is the ∆8 desaturase to convert EDA and/or EtrA to DGLA and/or ETA. The last two steps are the same between these two pathways.

It should be noted that the ∆15 desaturase and ∆17 desaturase are omega-3 desaturases; these two enzymes convert the omega-6 fatty acids into omega-3 fatty acids. The ∆15 desaturase converts LA to ALA. So far, there is no ∆15 desaturase found to convert LA to ALA with 100 % efficiency, so transformed cells with a heterologous ∆15 desaturase gene will contain both LA and ALA. Therefore, the ∆6 and ∆9 pathways can simultaneously use both LA and ALA as primary substrates. Apart from its primary function to convert ARA to EPA, most ∆17 desaturases can also convert EDA to ETrA with less efficiency.

All these desaturation and elongation enzymes carry out their reactions in the endoplasmic reticulum (ER) membrane (Meesapyodsuk and Qiu 2012). It is believed that the substrates of desaturases and elongases are in the form of phospholipid and acyl-CoAs, respectively. Elongation usually is the rate-limiting step of the aerobic pathways for EPA biosynthesis. Nevertheless, introduction of either the ∆6 or ∆9 pathway genes into the wild-type strain should allow the production of EPA through desaturation and elongation of the native fatty acid species.

## Toolbox for metabolic engineering of *Y. lipolytica* for EPA production

Before we started our project, the *Y. lipolytica* transformation system (Chen et al. 1997) had been established, and a low-copy replication plasmid had been developed (Fournier et al. 1993). Scientists were trying to use it as a host for efficient secretion of expressed heterologous proteins (Davidow et al. 1987a, b) or as a system to study certain biological functions. There were limited genetic elements such as promoters and terminators available that are necessary for metabolic engineering (Muller et al. 1998; Juretzek et al. 2001). We quickly found that the expression of an introduced gene was higher when it integrated into the genome than when it was in the low-copy replication plasmid. In order to develop *Y. lipolytica* as a host for EPA production by metabolic engineering, we pursued several research goals simultaneously.

1. Enrich the tool box for metabolic engineering
2. Development and test strategies for introducing multiple copies of foreign genes into *Y. lipolytica*
3. Establish a screening system for strain development
4. Develop the fermentation process

To demonstrate that *Y. lipolytica* can be engineered to produce EPA, we licensed the promoter of the translation elongation factor (TEF) gene of *Y. lipolytica* from Novozyme Corporation (Muller et al. 1998) and used it to drive the expression of the individual genes encoding ∆6 desaturase, C_18/20_ elongase, ∆5 desaturase, and ∆17 desaturase that we licensed from Ross Division of Abbott Laboratories (Knutzon et al. 1998; Picataggio et al. 2007). Integration of a single copy of these four gene expression cassettes into the genome of *Y. lipolytica* strain ATCC #20362 resulted in the synthesis of EPA at about 3 % of the total fatty acid methyl esters (FAME), with 34 % of all fatty acids derived from the engineered pathway, and the majority was GLA (Zhu et al. 2010). This result demonstrated that *Y. lipolytica* could be engineered to produce EPA and suggested that additional engineering improvements were needed to (1) increase the carbon flux into the engineered pathway, (2) improve the efficiency of the C_18/20_ elongases to convert more GLA into DGLA, and (3) enhance the expression of other pathway genes.

To enhance the expression of foreign genes in *Y. lipolytica*, we isolated a set of promoters that are at least as strong as the TEF promoter (Muller et al. 1998). The promoters we isolated are from the genes encoding export protein (*EXP*, YALI0C12034g), fructose 1,6-bisphosphate aldolase (*FBA1*; YALI0E26004g), glycerol-3-phosphate-*O*-acyltransferase (*GPAT*, YALI0C00187g), phosphoglycerate mutase (*GPM1*; YALI0B02728g), glycerol-3-phosphate dehydrogenase (*GPD1*; YALI0B02948g), and an ammonium transporter (*YAT1*, YALI0E27181g). All these promoter activities were compared with TEF promoter by quantitative fluorometric assays of the beta-glucuronidase (GUS) reporter (Jefferson et al. 1987) driven by each individual promoter. The results showed that the *FBA1* promoter was the strongest among the six promoters. The *GPM1* promoter was as strong as the *TEF* promoter, the *GPD1* promoter was 2.5 times stronger than the *GPM1* promoter, and the *FBA1* promoter activity was 5.5 and 2.2 times stronger than the *GPD1* and *GPM1* promoters, respectively.

In the N-terminal coding region of the *FBA1* gene, there is a 102 base pair intron located between the codons for amino acids 20 and 21. Fusion of the *FBA1* promoter plus the *N*-terminal coding region covering the first 23 amino acids and the intron (FBA1_in_) with the GUS reporter gene resulted in GUS activity about five times greater than the FBA promoter alone. As in the case of the *FBA1* gene, the *GPD1* gene also has an intron located in its N-terminus, which can significantly enhance the GPD promoter activity (Picataggio and Zhu 2007). The *N*-terminal coding regions with introns of *FBA1* and *GPD1* genes enhanced the activity of GPM promoter when chimeric promoters were constructed (Hong et al. 2011b).

The YAT1 promoter has a unique feature, since it has almost no activity under normal growth conditions, but under nitrogen-limiting conditions, its activity increased approximately 35-fold (Xue and Zhu 2012). This promoter is useful for directing the expression of omega-3 biosynthetic genes, because lipid synthesis and accumulation need nitrogen starvation. The relative strength of these promoters in nitrogen-limiting conditions was determined by quantitative GUS assays, and they are as follows: FBA_in_ > YAT1 > FBA > GPD, EXP > GPAT > GPM = TEF.

There are about 6,500 genes in *Y. lipolytica*. The promoter of each gene has a unique property. A diverse promoter library could also be generated through random mutagenesis using a specific promoter as template (Alper et al. 2005). Promoter characteristic studies are required before they can be utilized to maximize the expression of targeted genes in desired conditions.

**Fig. 3:**
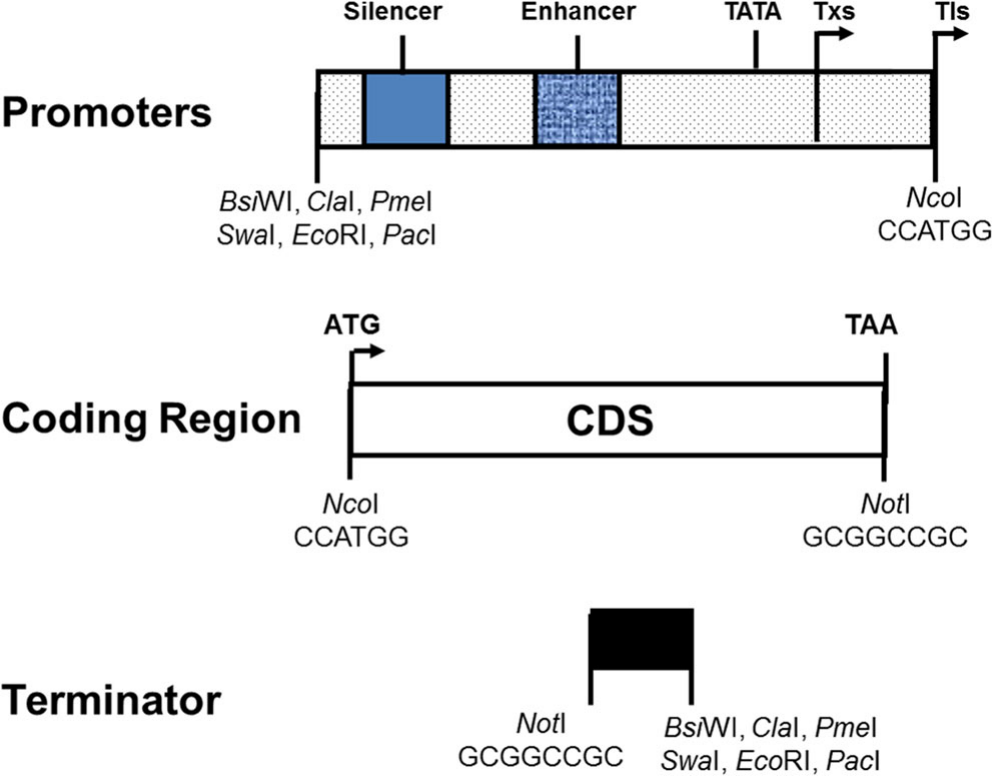
Modular genetic elements for chimeric gene construction

To improve the expression of foreign genes in *Y. lipolytica*, we developed a program to codon-optimize all the genes according to the codon usage pattern and GC content of highly expressed genes of *Y. lipolytica*. There are two ∆6-desaturase genes from *Mortierella alpina* and *Saprolegnia diclina*, two C_18/20_-elongase genes from *M. alpina* and *Thraustochytrium aureum*, three ∆5-desaturase genes from *Isochrysis galbana*, *M. alpina*, and *S. diclina*, and one ∆17-desaturase gene from *S. diclina* (Zhu et al. 2010, Knutzon et al. 1998, Picataggio et al. 2007). The optimized genes also contain the consensus sequence (5′-ACCATGG-3′) around the “ATG” translation initiation codon. We discovered that the substrate conversion was increased in almost all of the codon-optimized genes except the ∆5-desaturase gene derived from *M. alpina*. The improved substrate conversion efficiency of these “codon-optimized” genes is hypothesized to result from more efficient translation of their encoded mRNAs in *Y. lipolytica*. Right now, the genes introduced into *Y. lipolytica* for strain construction are all codon-optimized, and each of the synthetic genes were designed with an *Nco*I site around its translation initiation site and a *Not*I site after its stop codon. The modular structure of these coding regions, promoters, and terminators is easy to swap for construction of expression constructs with different configurations (Fig. 3).

**Fig. 4:**
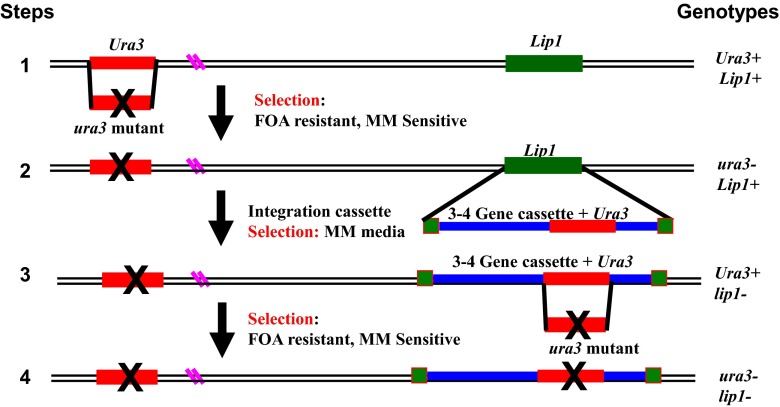
Strategy for integration of multiple copies of genes into the chromosomes of *Y. lipolytica. FOA* 5-fluoroorotic acid, *MM* minimal medium

From our early experiments, it was clear that we needed to introduce multiple copies of genes involved in EPA biosynthetic pathway for production of high amounts of EPA in engineered *Y. lipolytica* cells. Using the *URA3* gene and its counter selection by 5-fluoroorotic acid (FOA), we developed a system that can integrate many copies of foreign genes into the genome of *Y. lipolytica* via sequential integrations. FOA is toxic to yeast cells that possess a functional *URA3* gene, and this compound is not toxic to the yeast cells with an inactivated *ura3* gene (Barth and Gaillardin 1996; Zhu et al. 2010). As shown in Fig. 4, the native *URA3* gene can be knocked out by using a DNA fragment with mutated *ura3*, and the transformants growing on FOA plates will have the *ura3*-phenotype. A cluster of multiple chimeric genes (or a single chimeric gene) and a new *URA3* gene can be integrated into a different locus of the genome of *Y. lipolytica* thereby producing a new strain having an *URA3* ^+^ phenotype. Subsequent integration by homologous recombination with mutated *ura3* would produce a new *ura3* ^−^ strain, identified with FOA-resistant selection. Thus, the *URA3* gene (in combination with FOA selection) can be used as a selection marker in many rounds of transformation to introduce a large number of genes into the genome.

By increasing the copy number of the ∆6 pathway genes and “pushing” the carbon flux into the engineered pathway by overexpression of the C_16/18_-elongase gene of *M. alpina* (Macool et al. 2008) to convert the palmitic acid (C16:0) into stearic acid (C18:0) and the ∆12-desaturase gene of *Fusarium moniliforme* (Yadav et al. 2009) to convert the oleic acid to LA, we generated strain Y9027 to produce EPA at about 40 % of FAME (Zhu et al. 2010). This strain contained 19 copies of ten different heterologous genes that integrated into its genome. The in vivo substrate conversion catalyzed by the ∆6, ∆5, and ∆17 desaturases was about 86, 90, and 97 %, respectively, indicating that these enzymes functioned well in strain Y2097. The second highest fatty acid in the lipids was GLA, at about 21 % of FAMEs. The substrate conversion from GLA to DGLA catalyzed by C_18/20_ elongase was only about 69 % in that strain. The GLA is the product of ∆6 desaturase and the substrate of C_18/20_ elongase. The ∆6 desaturase introduces a double bond into the LA acyl chain esterified to a phospholipid backbone; the C_18/20_ elongase catalyzes the condensation of a malonyl group to GLA acyl chain esterified to coenzyme A (Domergue et al. 2003; Meesapyodsuk and Qiu 2012). There were four copies of C_18/20_-elongase genes driven by strong promoters in strain Y2097, suggesting that the C_18/20_-elongation reaction was a bottleneck in the engineered pathway. Thus, to reduce the GLA, amount in the lipids requires not only an efficient C_18/20_-elongase activity but also an active acyl exchange between the phospholipid and CoA pools in the ER membrane. To enhance the acyl-exchange process, we separately amplified genes encoding for acyl-CoA synthase (ACS), choline phosphotransferase (CPT1), diacylglycerol acyltransferase (DGAT1 and DGAT2), glycerol-3-phosphate acyltransferase (GPAT), lysophosphatidic acid acyltransferase (LPAAT), lysophosphatidylcholine acyltransferase (LPCAT), phospholipase C (PCL1), and phospholipase D (SPO22). These modifications each improved the GLA conversion efficiency up to 15 %. It is hypothesized that a combination of some of these genes could improve the efficiency of the C_18/20_ elongase, but it would not be able to eliminate the GLA, in engineered *Y. lipolytica* strains.

## Generation of EPA commercial production strains using the ∆9 pathway

In order to reduce the level of omega-6 fatty acids, especially the GLA, in lipids enriched with EPA, we decided to use the ∆9 pathway (Fig. 2). The selection of the ∆9 pathway ensured that the rate-limiting elongation is the first step of the engineered pathway; therefore, the accumulation of other intermediates should be kept to a minimum. The genes encoding Δ9 elongases (Damude et al. 2007) and Δ8 desaturases (Damude and Zhu 2007) were isolated and characterized from *Euglena gracilis*, *Euglena anabaena*, and *Eutreptiella*, sp. CCMP389. To increase the ∆8-desaturase activity, we also constructed three ∆9-elongase and ∆8-desaturase bifunctional fusion genes (Damude et al. 2008); the Δ8-desaturase activity in these fusion enzymes increased almost 100 % compared with ∆8-desaturase alone while keeping similar ∆9-elongase activity. At the same time, three genes encoding ∆5 desaturases from *E. gracilis*, *E. anabaena*, and *Eutreptiella*, sp. CCMP626 (Pollak et al. 2012), and three genes encoding Δ17 desaturases from *Pythium aphanidermatum*, *Phytophthora ramorum*, and *Phytophthora sojae* were also isolated and studied for their activities and substrate selectivity (Xue et al. 2013b). Additionally, several genes encoding different acyltransferases (Zhang et al. 2012) were also isolated and used to improve fatty acid traffic in the ER.

To construct a high EPA production strain using the Δ9 pathway, we used a series of strategies. First, an efficient EPA biosynthetic pathway was built by using strong promoters such as EXP1, FBAINm, GPAT, GPD, and YAT, all heterologous genes were codon-optimized and multiple copies of structural genes were inserted for each step. To increase the ∆8-desaturase activity, several copies of ∆9-elongase and ∆8-desaturase bifunctional fusion genes were employed (Damude et al. 2008). Second, “pushing” and “pulling” the carbon flux into the engineered ∆-6 pathway were achieved by overexpression of the C_16/18_-elongase gene (Macool et al. 2008) and the ∆12-desaturase gene (Yadav et al. 2009) and by using multiple copies of ∆17-desaturase genes (Xue et al. 2013b). Third, beta-oxidation was reduced by knockout of genes encoding for peroxins (Xue et al. 2013a; Hong et al. 2009) such as *PEX3* or *PEX10* that are involved in peroxisome biogenesis and matrix protein import. Fourth, fatty acid transport was controlled by fine regulation of different acyltransferases to increase fatty acid flux for EPA production and lipid accumulation.

Since *Y. lipolytica* strain ATCC #20362 prefers nonhomologous end-joining over homologous recombination (Weterings and Chen 2008), we designed to screen 96 transformants for each parent strain/construct combination by gas chromatography (GC) analysis (Cahoon et al. 2001). The nonhomologous recombination arising from each transformation generated a library of transformants with diverse performance. Several beneficial traits for EPA and lipid production were found through screening this diversity. For example, knockout of the *PEX10* gene (Xue et al. 2013a; Hong et al. 2009) was discovered to increase EPA titer in lipid to more than twice as those of its PEX+ siblings. We also discovered that deletion of the *PEX10* gene in DGLA and ARA production strains could also more than double DGLA and ARA titers in lipids compared to the parent strains with a wild-type *PEX10* gene (Xue et al. 2013a). In these strains with *pex10*∆, beta-oxidation is greatly reduced and there were no normal peroxisomes inside cells. Unidentified membrane-like structures are observed that might be the deformed nonfunctional peroxisomes.

By combining the above strategies, we first generated the Gen I strain Y4305 (Xue et al. 2013a) that contains 30 copies of 9 different genes and produces EPA at 56.6 % FAME, without GLA accumulation. The total EPA produced was about 15 % of DCW. The lipid produced by strain Y4305 has a unique and healthy fatty acid profile that contains less than 5 % saturated fatty acids and has only small amounts of intermediates. As is the case in strain Y2097 using the ∆6 pathway, the substrate conversion efficiency of the introduced desaturases is still significantly higher than the elongases in strain Y4305. However, the selection of the ∆9 pathway ensured that the rate-limiting elongation is the first step of the engineered pathway. Accumulation of intermediates is therefore minimized, in contrast to cells engineered with the ∆6 pathway where the first step is not rate limiting and accumulation of GLA becomes significant.

To increase the rate, titer, and yield for EPA production, we generated Gen II strain Z1978 (Hong et al. 2011a) via 24 steps of genetic modifications. It contained 35 copies of 17 different genes and produces EPA at >58 % FAME. The total EPA content in strain Z1978 is about 20 % DCW. The fatty acid profile of strain Z1978 is similar to that of strain Y4305, with extremely low saturated fatty acids and only small amounts of intermediates and no GLA. Strain Z1978 has increased lipid contents over strain Y4305 from about 30 % DCW in Y4305 strain to more than 38 % DCW in strain Z1978.

Based on strain Z1978, we generated strain Gen III HP Z5567 (Hong et al. 2011a) by six more steps of metabolic engineering. Strain Z5567 contained 41 copies of 19 different genes. It produced EPA at about 50 % FAME but with a total lipid of more than 50 % DCW; therefore, strain Z5567 produced EPA at about 25 % DCW, which is about 25 and 67 % improvement over strain Z1978 and Y4305, respectively.

## Selection of production strains under fermentation conditions

**Fig. 5:**
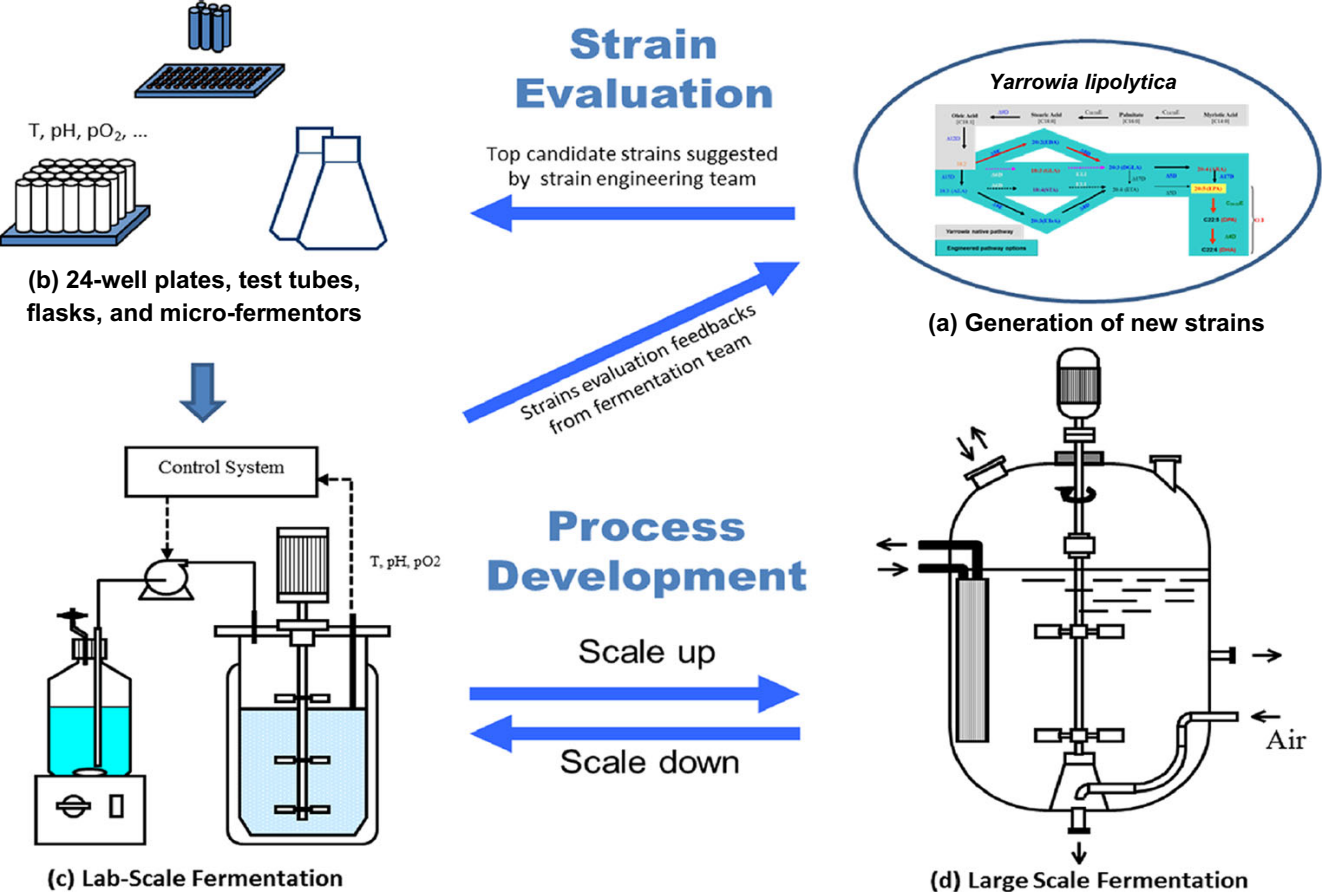
Workflow of omega-3 fermentation for both strain evaluation and process development. New strains generated by metabolic engineering (**a**) are sequentially evaluated in 24-well blocks, shake flasks, and micro-fermentors (**b**) before they are tested in lab-scale fermenters (**c**). Strains that perform well in lab-scale fermentors are further tested in pilot scale (**d**) before being adopted for EPA production in commercial-scale fermenters

Fermentation process development as well as strain engineering plays a critically important role in converting the fundamental research to real commercial application. The fermentation research was initiated at the start of the strain engineering research. Fermentation experimentation included (1) strain screening under fermentation conditions, (2) optimization of fermentation conditions for each promising new strain, and (3) process scale-up. Figure 5 summarizes the typical workflow for the omega-3 fermentation research work. Figure 5a represents the generation of candidate strains by metabolic engineering in *Yarrowia* cells. Thousands of new or promising strains that are generated by metabolic engineering were tested sequentially in small-scale simple bioreactors (working volume = 1 ~ 100 mL), which included 24-well blocks, test tubes, shake flasks, and micro-fermentors (Fig. 5b). The omega-3 fermentation is a two-stage (growth + lipid production/oleaginous) process. Therefore, cells are first grown in these simple bioreactors to a specified density after which they are deprived of nitrogen for growth and given glucose for maintenance and lipid production. At the end of the production stage, the lipid composition in the *Yarrowia* biomass, including EPA and other fatty acids, is determined by GC analysis (Cahoon et al. 2001). In shake flask and micro-bioreactor experiments, dry cell weight (DCW) is also determined due to the availability of more sample volume (~5 mL), so that the total lipid content of each sample (total lipid/DCW) is determined to better evaluate the biomass, lipid, and EPA productivities.

Dozens to hundreds of candidate strains that show high EPA content in lipid and high lipid content in biomass in the simple bioreactors are taken one step further to lab-scale fermenters (2 ~ 10 L), in which the dissolved oxygen, pH value, and especially substrate feed can be controlled more consistently than is possible in the simple bioreactors. The lab-scale fermentation results, including the time course data for EPA titer, rate, and yield, are used to determine the very top strains to be scaled up in pilot-scale fermentors under commercially achievable medium and process conditions (Fig. 5d).

The rate-limiting step in the workflow shown in Fig. 5 is the use of some simple bioreactors (24-well blocks, test tubes, flasks, and micro-fermentors) to identify the top strains or to prescreen medium and process conditions (Xie 2012). It is desirable to have a multi-bioreactor system with small working volumes to allow testing of thousands of candidate strains, yet each small bioreactor system must have high-quality process and feed controls so that the data obtained from these small bioreactors predict the performance in lab- and pilot-scale fermentors.

The 24-well block or test tube is the simplest bioreactor, but its data is much less reliable due to the low controllability in a small volume and insufficient sample volume available for both lipid content and lipid composition analysis (Danielson et al. 2004; Stockmann et al. 2003). Shake flasks are also simple and easy to run at larger working volumes (10 ~ 100 mL) typically without monitoring and controlling pH values and dissolved oxygen (DO) levels, but they are relatively labor intensive to prepare and require fairly bulky shakers (Büchs 2001). Micro-titer plates/bioreactors have a large number of small reaction wells (1 mL or less) with each well’s pH value and DO level possible to monitor, which is very efficient for high-throughput growth evaluation (Duetz et al. 2000; Amanullah et al. 2010). However, the precise controls of pH values and DO levels are still not available for most micro-titer/bioreactors on the market. The small working volume also limits the micro-titer bioreactor’s application in the omega-3 project due to the insufficient biomass samples (less than 5 mL) available for DCW and total lipid content analysis.

**Fig. 6:**
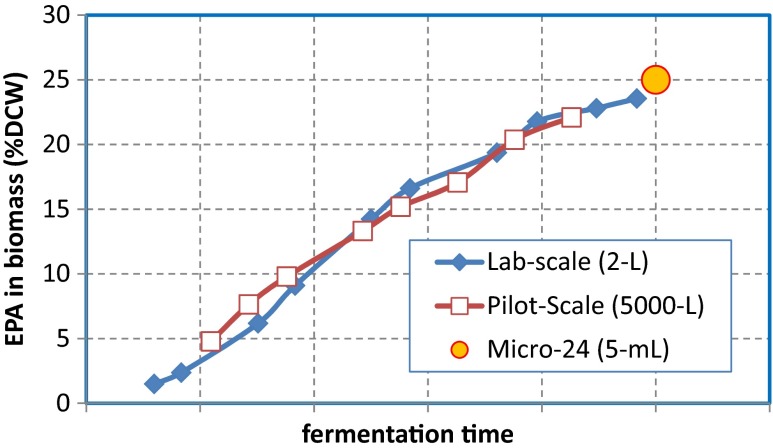
EPA titer comparison of Gen III HP strain in typical runs of micro-24 bioreactor (5 mL), lab-scale fermentor (2 L), and pilot-scale fermentor (5,000 L). The micro-fermentor achieved very consistent performance compared with the larger-scale fermentation

To minimize the limitations in control and to combine the high-throughput advantages of the 24-well blocks, test tubes, flasks, and micro-titer/bioreactors, we used an advanced micro-fermenter system for EPA strain screening and fermentation optimization (Xie 2012). A micro-fermenter is a highly integrated multi-fermentor system with a few milliliters of working volume per reactor; each reactor was independently controlled at the preset temperature, pH value, and DO level. There have been several different types of micro-fermenters reported in fermentation research work (Doig et al. 2005; Betts et al. 2006; Gilla et al. 2008). We used a Micro-24 bioreactor system for the EPA strain screening (Xie 2012). In this case, 24 individual micro-reactors, each with its own strain and process condition (T, pH, DO), can be processed at the same time. The high controllability of this system provided high-quality fermentation data for the end-of-run samples, including the by-product analysis, DCW, lipid content, EPA content in lipid, and EPA conversion yield. These data significantly increased the predictability of each individual strain’s performance in lab- or large-scale fermentation, as shown in an example in Fig. 6.

## Optimization of lab-scale fermentation

**Fig. 7:**
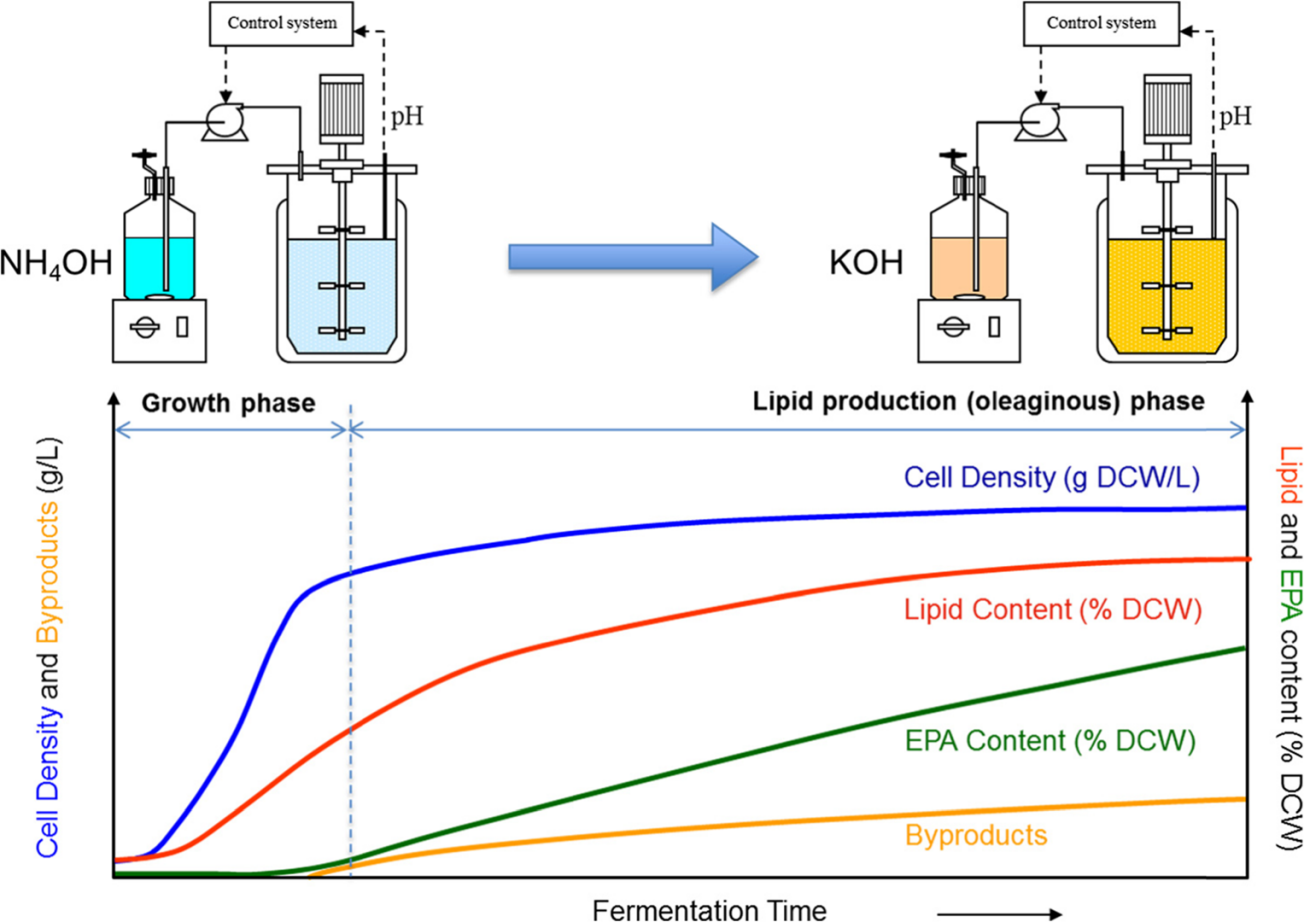
EPA production by a two-stage fermentation developed primarily in lab-scale fermenters. In growth phase, nitrogen is mainly provided by NH_4_OH for pH control to build up biomass. During the production phase, nitrogen is limited by switching the ammonium base to KOH to produce lipid with high EPA content

After we selected the top candidate strains by micro-fermentation analysis, we needed to further optimize the medium and process conditions (e.g., T, pH, DO, substrate feed) for a given strain to maximize its EPA production and minimize by-product formation (e.g., organic acids), in order to reduce the cost of manufacture (COM) under commercially achievable conditions. While flasks and micro-fermenters gave some guidance around optimization of the fermentation conditions, they are not sufficient for understanding, exploring, and further improving a complete fermentation run’s titer, rate, yield, and cost of a fermentation process at large scale. For that purpose, the optimization work for a selected production strain was mainly conducted in lab-scale fermenters. Since the EPA lipid is an intracellular product of the *Yarrowia* biomass, the goal of optimization is first to maximize the biomass production in growth phase, and then to maximize EPA production and minimize by-product formation in the oleaginous phase. We developed a two-stage (or two-phase) fed-batch fermentation process to maximize both biomass and EPA production, as shown in Fig. 7. In the first stage of the fermentation, the *Yarrowia* cells are grown on the carbohydrate substrate with nitrogen provided by both the organic nitrogen source (e.g., yeast extract) in the nutrient medium and the inorganic NH_4_OH used for pH control. After a certain period of time, the base for pH control is switched from NH_4_OH to KOH to restrict further nitrogen supply. Cell growth then stops after consuming the residual nitrogen in the medium, and the *Yarrowia* cells start to accumulate lipids by using the carbohydrate supplied. Our experience showed that the optimal conditions often vary when the strain is engineered in significantly different genetic backgrounds. Therefore, more optimization work is always expected to improve a newly selected strain’s performance.

## Using modeling to guide fermentation process optimization and scale-up

**Fig. 8:**
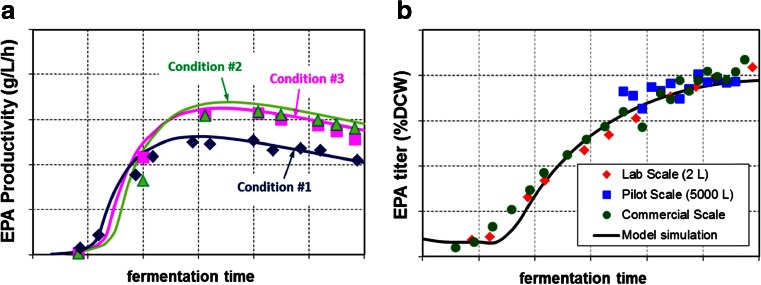
Examples of using the predictive fermentation model for process optimization (**a**) and scale-up (**b**). **a** The model simulation (*solid lines*) guided the experiments (*symbols*) to significantly improve EPA productivities by changing the process condition from *#1* to *#2* and *#3*. **b** The model (*solid line*) was used to design the scale-up fermentation conditions so that very comparable results (*symbols*) were achieved in lab-, pilot-, and commercial-scale fermentors

Though the optimization of lab-scale fermentation is critical for a selected production strain to achieve good performance at large scale, it is very time-consuming and labor-intensive. As we had accumulated more fermentation data and gained more understanding for both the strain and process, we used a set of mathematical equations, i.e., the dynamic models, to describe the fermentation behavior. A unique and also critical aspect of process development and scale-up for the EPA project was the use of dynamic models. Unstructured mathematical models were built from first principles, which included the model equations of cell growth, substrate consumption, nitrogen utilization, oxygen uptake, lipid and EPA formation, and by-product accumulation. The models were matched to the historical experimental data from many lab-scale and pilot-scale fermentation experiments under different conditions. The models could predict cell growth, DCW, DO level, oxygen uptake rate (OUR), CO_2_ evolution rate (CER), and variables that were also measured during the fermentation as a function of various medium and process conditions. When a new production strain was applied, a few model parameters may need to be adjusted to keep the model’s predictability based on the new experimental data and the understanding of the new strain. The dynamic models were thus able to predict the key performance parameters (e.g., titer, rate, and yield of a product) before and during the run and further help guide the fermentation optimization and process scale-up. Examples of the dynamic model’s capability are shown in Fig. 8.

## Fermentation scale-up

The last step in fermentation process development is the scale-up to pilot and then commercial scales. There are a few criteria commonly used for fermentation scale-up, including geometry similarity, power input, and mass transfer coefficient *K*_*La*_ (Shuler and Kargi 2002). For the EPA fermentation process, we often tested our selected production strain in the pilot-scale facilities before we set a criterion for scale up, by which we were able to gather information of the pilot-scale fermentation’s dynamic behavior. Besides the regular online data of temperature, pH value, feed rate, and DO levels, the dynamic information also included the agitation, aeration rate, and the mass transfer characteristics. Now, the benefits of highly predictive dynamic models became even more evident as we moved from the pilot plant to commercial production. We incorporated the agitation and aeration rate of the pilot-scale fermentation into the dynamic models so that we could study mass transfer characteristics as functions of superficial gas velocity and agitation power for each run. We then used the dynamic models to predict the commercial-scale fermentation’s performance with the specific agitation and aeration in commercial-scale fermentors and thus guided the successful scale-up for the commercial production.

However, we quite often faced some challenges when there were some restrictions in the commercial scale fermentors, either for the use of some important medium components or for the process control that we could achieve at commercial scale. For example, the commercial scale fermentor uses only commercially available raw materials, which may have some other minor components affecting fermentation performance. Also, the much larger size of a commercial-scale fermentor causes significantly different fluid dynamic behavior in the reactor. To understand how a difference of a commercial-scale fermentor affects the fermentation results, we designed a series of lab-scale experiments to mimic the fermentation with the medium and/or process conditions at commercial scale. These are called “scale-down” studies (Ozbek 1997; Nienow et al. 2011). By the scale-down studies, we were able to identify a few important factors that affecting the scale-up. Then, we either sent the feedback to the strain-engineering team to reengineer the strain or modified our fermentation protocols for the lab-scale and pilot-scale experiments to fix the possible scale-up problems, as previously indicated in Fig. 5.

## Conclusions

The *Y. lipolytica* yeast was engineered in DuPont to produce a high level of EPA in biomass under commercial-scale fermentation conditions. By overexpressing a combination of enzymes that are necessary for synthesis of EPA via the ∆9/∆8 pathway and for optimization of lipid metabolism, the Gen III HP strain was created that is capable of making EPA at 25 % DCW and at more than 50 % lipids. The high level of EPA production was achieved through careful balancing of the expression levels of various pathway enzymes, and modification of fatty acid and lipid metabolism of the host. Disruption of the peroxisome biogenesis gene had a major positive impact on the production of EPA and the metabolism of storage lipid, as well as reduction of the major by-products.

Both research studies in strain engineering and fermentation process development were initiated at the same time to help convert the lab research results to commercial production. Advanced micro-fermentors with well-controlled process parameters significantly improved the efficiency of strain screening and the predictability of the selected strains’ fermentation performance at much larger scale. A two-stage fed-batch fermentation process was developed to maximize both biomass and EPA production and minimize the by-product formation. Finally, mathematical modeling of the developed fermentation process and scale-down studies played critically important roles in the successful process scale-up. Two commercial products, New Harvest™ EPA oil and Verlasso® salmon, were developed using our sustainable EPA source. Our journey demonstrated the power of modern biotechnology by combining both fundamental scientific research and industrial engineering.
